# Salvage chemoradiotherapy after primary chemotherapy for locally advanced pancreatic cancer: a single-institution retrospective analysis

**DOI:** 10.1186/1471-2407-12-609

**Published:** 2012-12-20

**Authors:** Hiroshi Mayahara, Yoshinori Ito, Chigusa Morizane, Hideki Ueno, Takuji Okusaka, Shunsuke Kondo, Naoya Murakami, Madoka Morota, Minako Sumi, Jun Itami

**Affiliations:** 1 Division of Radiation Oncology, National Cancer Center Hospital, 5-1-1 Tsukiji, Chuo-ku, Tokyo, 104-0045, Japan; 2 Divisions of Hepatobiliary and Pancreatic Oncology, National Cancer Center Hospital, 5-1-1 Tsukiji, Chuo-ku, Tokyo, 104-0045, Japan

**Keywords:** Pancreatic cancer, Locally advanced pancreatic cancer, Induction chemotherapy, Salvage therapy, Chemoradiotherapy, Prognostic factor

## Abstract

**Background:**

There is no consensus on the indication for salvage chemoradiotherapy (CRT) after failure of primary chemotherapy for locally advanced pancreatic cancer (LAPC). Here we report on the retrospective analysis of patients who received salvage CRT after primary chemotherapy for LAPC. The primary objective of this study was to evaluate the efficacy and safety of salvage CRT after primary chemotherapy for LAPC.

**Methods:**

Thirty patients who underwent salvage CRT, after the failure of primary chemotherapy for LAPC, were retrospectively enrolled from 2004 to 2011 at the authors’ institution. All the patients had histologically confirmed pancreatic adenocarcinoma.

**Results:**

Primary chemotherapy was continued until progression or emergence of unacceptable toxicity. Eventually, 26 patients (87%) discontinued primary chemotherapy because of local tumor progression, whereas four patients (13%) discontinued chemotherapy because of interstitial pneumonitis caused by gemcitabine. After a median period of 7.9 months from starting chemotherapy, 30 patients underwent salvage CRT combined with either S-1 or 5-FU. Toxicities were generally mild and self-limiting. Median survival time (MST) from the start of salvage CRT was 8.8 months. The 6 month, 1-year and 2-year survival rates from the start of CRT were 77%, 33% and 26%, respectively. Multivariate analysis revealed that a lower pre-CRT serum CA 19–9 level (≤ 1000 U/ml; *p* = 0.009) and a single regimen of primary chemotherapy (*p* = 0.004) were independent prognostic factors for survival after salvage CRT. The MST for the entire patient population from the start of primary chemotherapy was 17.8 months, with 2- and 3-year overall survival rates of 39% and 22%, respectively.

**Conclusions:**

CRT had moderate anti-tumor activity and an acceptable toxicity profile in patients with LAPC, even after failure of gemcitabine-based primary chemotherapy. If there are any signs of failure of primary chemotherapy without distant metastasis, salvage CRT could be a treatment of choice as a second-line therapy. Patients with relatively low serum CA19-9 levels after primary chemotherapy may achieve higher survival rates after salvage CRT. The strategy of using chemotherapy alone as a primary treatment for LAPC, followed-by CRT with salvage intent should be further investigated in prospective clinical trials.

**Trial registration:**

2011–136

## Background

The prognosis of pancreatic cancer remains dismal. The 5-year overall survival of patients with pancreatic cancer is < 5%. In Japan, about 27,000 patients are estimated to have pancreatic cancer, and almost the same numbers of deaths annually are attributable to this cancer. Although surgical resection offers the opportunity for cure, less than 20% of patients are diagnosed with pancreatic cancer at an early resectable stage. At initial diagnosis, ≥ 80% of patients with pancreatic cancer have locally advanced or metastatic disease.

Locally advanced pancreatic cancer (LAPC) is defined as surgically unresectable disease without detectable metastases. Historically, concurrent chemoradiotherapy (CRT) with 5-fluorouracil (5-FU) has been the standard treatment since it offers survival benefit when compared with best supportive care [1], radiotherapy alone [2] and chemotherapy with 5-FU alone [3]. Recently, 5-FU has been replaced by oral fluorouracil analogues such as S-1 in East Asia [4] and capecitabine in Western countries. When taken orally these drugs are much more convenient to administer than 5-FU, which usually requires protracted venous infusion. S-1 is an oral agent that contains tegafur, gimeracil and oteracil in a molar ratio of 1:0.4:1 [5]. S-1 is reported to be at least equivalent to or even more active than 5-FU when combined with radiotherapy for LAPC [6-8].

The standard method used for the detection of metastases from pancreatic cancer is computed tomography (CT). Several investigators have reported that intraoperative staging can reveal occult peritoneal dissemination in 6–37% of the patients with CT-diagnosed LAPC [9-11]. Analysis of patterns of failure after definitive CRT for LAPC has shown that more than half of the patient will have distant metastasis at the first time of failure [12]. Because radiotherapy involving the primary site offers little benefit to patients with occult distant metastasis, increasingly more oncologists believe that chemotherapy would be a preferable initial therapeutic approach for patients with LAPC [13]. During ini-tial chemotherapy, rapidly progressive chemotherapy-resistant distant metastases will present within a few months. After 3–6 months of induction chemotherapy, LAPC that remained local would be an indication for consolidative or salvage CRT. However, there is no consensus on the indications for additional CRT following primary chemotherapy for LAPC, as well as the optimal time period for the administration of primary chemotherapy. Here we report on the results of a retrospective analysis of this strategy, including primary chemotherapy and salvage CRT, for patients with LAPC. The primary objective of our study was to evaluate the efficacy and safety associated with salvage CRT following primary chemotherapy for LAPC. The secondary objective was to elucidate the prognostic factors that affect survival after CRT.

## Methods

### Patients

Between October 2004 and August 2011, 98 patients who were diagnosed as having LAPC underwent CRT at the author’s institution. Sixty-seven patients were excluded from the study because they had received definitive CRT as the first therapeutic modality. One patient was excluded because he had undergone consolidative CRT after primary chemotherapy. The remaining 30 patients underwent salvage CRT after the failure of primary management with chemotherapy alone. All of the patients had histologically confirmed pancreatic adenocarcinoma. They were subjected to intensive analysis. The clinical data from these patients were entered into the database in September 2012. Our institutional review board (Institutional Ethical Review Board of the National Cancer Center) approved this study.

### Treatment strategy

At the first diagnosis, multidetector row CT involving the chest and abdomen were performed for the assessment of the local extension of the primary tumor, and for excluding distant metastases. CT based criteria regarding tumor unresectability included encasement or occlusion of the celiac trunk, common hepatic artery, superior mesenteric artery or aorta. All of the patients with obstructive jaundice underwent biliary drainage prior to treatment.

Until December 2007, primary management with CRT combined with 5-FU was the principal treatment of choice for patients with LAPC [14]. Since 2006, several prospective phase II clinical trials involving patients with LAPC were conducted at the authors’ institution [4,8,15,16]. CRT combined with S-1 has been regarded as an optional treatment of choice in Japan [7,8]. A multi-institutional phase II trial with gemcitabine (GEM) alone for LAPC yielded promising results with a low toxicity profile [15]. Additionally, our retrospective study revealed that there was no difference in the survival rates of the patients who received CRT or GEM-based chemotherapy alone as a primary therapy for LAPC [17]. Although direct comparison between primary CRT and primary chemotherapy alone has not yet been made in a prospective clinical trial, GEM monotherapy has been regarded as the first treatment of choice in clinical practice since January 2008.

Currently, all of the patients with LAPC are informed of two first-line treatments of choice, namely GEM monotherapy and CRT combined with S-1. If a patient with LAPC has an indication suitable for participation in a clinical trial, the patient will be given additional information about that trial. The patients themselves selected one of these treatments. The current study included patients who initially entered prospective clinical trials involving primary chemotherapy and who subsequently received CRT as a salvage treatment.

### Eligibility criteria for salvage CRT

Indications for salvage CRT following chemotherapy included the following: no distant metastasis; no prior radiotherapy of the upper abdomen; Karnofsky performance status (KPS) ≥ 70; adequate hematologic function (leucocyte count ≥ 3,500/μL and platelet count ≥ 100,000/μL ); and hepatic function (bilirubin ≤ 2.0 mg/dL, aspartate aminotransferase (AST)/alanine aminotransferase (ALT) ≤ 150 U/L) and renal function (serum creatinine < 1.5 mg/ml). The exclusion criteria were the presence of: an active gastroduodenal ulcer; watery diarrhea; ascites; active infection; or mental disorder. Written informed consent was obtained from each patient before starting each treatment.

### First-line chemotherapy

Primary chemotherapy was continued until disease progression, the emergence of unacceptable toxicity or a patient’s refusal of treatment. First-line chemotherapy mostly consisted of GEM alone [Table 1]. GEM was administered intravenously at a dose of 1,000 mg/m^2^ over 30 min on days 1, 8 and 15, and was repeated every 4 weeks as one course. Patients with grade 3–4 hematological toxicities underwent dose reduction to 800 mg/m^2^ or skipped at least one administration of GEM. Prophylactic granulocyte-colony stimulating factor support was not used.

**Table 1:** Patient characteristics (n = 30)

** Characteristic**	**No. of patients**	**% patients**
Age (years)		
Median (range)	65 (42–81)	
Gender		
Male	16	53
Female	14	47
Karnofsky performance status		
90-100	22	73
70-80	8	27
0-60	0	0
Tumor location		
Head	15	50
Body and Tail	15	50
Nodal status		
Negative	18	60
Positive	12	40
Baseline tumor diameter (cm)		
Median (range)	4.5 (2.1-7.8)	
Baseline serum CA19-9 level (U/ml)		
Median (range)	872 (0–35490)	
≥ 1,000	14	47
100-1,000	11	37
< 100	5	17
Pre-CRT tumor diameter (cm)		
Median (Range)	4.1 (1.9-8.4)	
Pre-CRT serum CA19-9 Level (U/ml)		
Median	631 (0–50440)	
≥ 1,000	11	37
100-1,000	12	40
< 100	7	23
Regimens of primary chemotherapy		
Gemcitabine alone	24	80
Gemcitabine + α	6	20

*CRT* chemoradiotherapy.

### Chemoradiotherapy

A planning CT was required to determine target volumes on the three-dimensional treatment planning system. A total dose of 50.4 Gy was delivered in 28 fractions using a linear accelerator of energy ≥ 10 MV. The clinical target volume (CTV) included the gross primary tumor and metastatic lymph nodes only. Elective nodal irradiation was not applied in this cohort. The planning target volume (PTV) was defined as the CTV plus 1 cm in all directions and a 1.5-2.0 cm margin in the craniocaudal direction to account for respiratory organ motion. The dose was prescribed to the center of the PTV. Typically, a 4 or 5 field technique was used to minimize high-dose radiation exposure in the surrounding organs.

Radiotherapy was delivered concomitantly with either 5-FU or S-1. Protracted 5-FU infusion was mainly administered until July 2008, and oral S-1 was given thereafter. Concomitant 5-FU was administered as a protracted venous infusion at a dose of 200 mg/m^2^/day from days 1–5 each week during the course of radiotherapy [14]. S-1 was administered orally twice daily after breakfast and dinner on weekdays (Monday through Friday) during irradiation. The standard dose of S-1 with concurrent radiotherapy for LAPC was 80 mg/m^2^/day [4]. Maintenance chemotherapy with S-1 was indicated for patients without obvious clinical progression during CRT, with sufficient performance status and organ function.

### Response and toxicity assessment

All of the medical charts of the eligible patients were reviewed. Information on potential prognostic factors was collected and included: age; gender; performance status; tumor diameter; change in serum carbohydrate antigen 19–9 (CA19-9) level; and sequence of treatments. Contrast-enhanced CT was performed before starting every two cycles of primary chemotherapy, before and at the end of CRT, and every 2 months after CRT. Objective tumor response was evaluated radiologically according to the Response Evaluation Criteria in Solid Tumors (RECIST) version 1.1 [18]. CA19-9 was continuously measured once per month. Toxicities were prospectively recorded at each patient’s visit using the Common Terminology Criteria for Adverse Events (CTCAE) version 3.0. The highest grades of toxicity observed during CRT and after CRT were recorded.

### Statistical analysis

Overall survival from the start of primary chemotherapy and salvage CRT was estimated using the Kaplan-Meier method. Times to progression at the primary tumor site or distant sites were also calculated. Progression was defined as confirmation of progressive disease on CT images using the RECIST criteria. For univariate and multivariate analysis, all of the variables were dichotomized according to clinical relevance based on the previous literature. Univariate analyses were performed using the log-rank test. A Cox’s proportional hazards model was developed to identify significant factors influencing survival after CRT. Possible confounded variables were excluded from multivariate analysis. All of the tests of hypotheses were conducted at an alpha level of 0.05 with a 95% confidence interval (CI). All of the statistical analyses were performed using SPSS Statistics version 17.0 (SAS Institute, Tokyo, Japan).

## Results

### Patient characteristics

Thirty patients with LAPC received primary chemotherapy and salvage CRT. The patient characteristics are summarized in [Table 1]. For first-line chemotherapy, all of the patients received GEM-based chemotherapy. GEM-based chemotherapy included GEM alone in 24 patients (80%) and GEM-based combination chemotherapy in six patients (20%).

### Sequel of first-line chemotherapy

The median number of cycles of GEM in 24 patients who received GEM monotherapy was six (range, 1–41). Best tumor response assessed radiologically and best CA19-9 response to first-line chemotherapy are summarized in Table 2. A partial response (PR) was achieved in nine patients, with a response rate of 30%. Among 24 patients whose baseline serum CA19-9 level was >100 U/ml, the median CA19-9 level decreased from 1151 U/ml at baseline to 159 U/ml at minimum during first-line chemotherapy. In these patients, the CA19-9 level decreased by ≥ 50% in 21 patients (88%); the median time to reach the minimum CA19-9 level was 4.0 (range, 1.8-13.0) months. After failure of first-line GEM-based chemotherapy, seven patients (23%) proceeded to second-line chemotherapy with S-1 alone. The median duration of continuing second-line chemotherapy was 3.0 months.

**Table 2:** Best response to primary chemotherapy

** Tumor response**	**No. of patients**	**% patients**
Radiological response		
Partial response	9	30
Stable disease	19	63
Progressive disease	2	7
CA19-9 response (base line CA19-9 > 100 U/ml)		
≥ 50% decrease	21	88
< 50% decrease	1	4
Increase	2	8

Eventually, 26 patients (87%) discontinued primary chemotherapy because of local tumor progression, whereas four patients (13%) discontinued chemotherapy because of interstitial pneumonitis caused by GEM. The reasons for discontinuation of the primary chemotherapy are summarized in Table 3.

**Table 3:** The reasons for discontinued primary chemotherapy

** Reason**	**No. of patients**	**% patients**
Presence of any types of primary disease progression (n = 26)		
Enlargement of tumor	14	47
Elevation of tumor marker	7	23
Carcinomatous pain	5	17
Obstructive jaundice	5	17
Duodenal hemorrhage	2	7
Absence of disease progression (n = 4)		
Interstitial pneumonia	4	13

### Sequence of salvage CRT

Thirty patients started salvage CRT after the failure of the primary chemotherapy. The median time between the start of the primary chemotherapy and the start of CRT was 7.9 (range, 3.0-37.3) months. All of the patients completed the course of radiotherapy without major interruption. The median duration of CRT was 42 (range, 38–45) days. Administration of the combined chemotherapeutic agents involved protracted infusion of 5-FU in 14 patients (47%) and oral S-1 in 16 patients (53%). Toxicities during and after CRT are listed in Table 4. Hematological toxicity was relatively mild and there was no grade 4 toxicity. The most frequent grade 3 hematological toxicity was leucopenia. Grades 3 and 4 non-hematological toxicity included anorexia (19%), nausea (6%), fatigue (6%), gastrointestinal ulcer (6%), vomiting (3%) and bile duct infection (3%). After CRT, three patients developed a gastrointestinal ulcer; of these, two (grade 2) recovered after conservative treatment, and one (grade 3) required endoscopic hemostasis. Another patient developed a duodenal fistula involving the primary tumor at 2 months after completion of CRT (grade 4). This fistula was possibly caused by the necrosis of the huge primary tumor that penetrated the duodenal wall. Although the hemorrhage was transient, this patient needed to undertake long-term fasting and intravenous hyperalimentation, but later died of severe bile duct hemorrhage because of primary tumor progression.

**Table 4:** Toxicity during and after salvage chemoradiotherapy

** Toxicity**	**Grade 0**	**Grade 1**	**Grade 2**	**Grade 3**	**Grade 4**	**Toxicity of any grade (%)**	**Toxicity of grade 3–4 (%)**
Hematological toxicity							
Leukopenia	6	11	11	3	0	81	10
Neutropenia	12	13	5	1	0	61	3
Anemia	4	14	10	3	0	87	10
Thrombocytopenia	12	16	3	0	0	61	0
AST/ALT	20	9	2	0	0	35	0
Non-hematological toxicity							
Fatigue	7	17	5	2	0	77	6
Anorexia	4	18	3	5	1	87	19
Nausea	9	15	5	2	0	71	6
Vomiting	24	6	0	1	0	23	3
Diarrhea	21	8	2	0	0	32	0
Abdominal pain	20	9	2	0	0	35	0
Stomatitis	29	2	0	0	0	6	0
Skin rash	29	2	0	0	0	6	0
Infection	29	0	1	1	0	6	3
Gastrointestinal ulcer	27	0	2	1	1	13	6

*AST* aspartate transaminase, *ALT* alanine transaminase.

Four patients were diagnosed as having distant metastasis immediately after the completion of salvage CRT. Because of poor general health and/or the lack of an efficacious chemotherapeutic regimen, these patients did not undergo further evaluation. The response of the primary tumor was evaluated radiologically at 2 months after the completion of CRT in 26 patients. Tumor response to CRT included a PR in one patient (3%), stable disease (SD) in 22 patients (73%) and progressive disease (PD) in three patients (10%). Among the 24 patients whose initial CA19-9 level was >100 U/ml, the median CA19-9 level decreased from 769 U/ml to 479 U/ml at minimum after CRT. The CA19-9 level decreased more than 50% in 14 patients (58%) after CRT. Relief of pain was achieved in 16 out of 19 patients (84%) who had experienced carcinomatous pain before CRT. After the completion of salvage CRT, 20 patients (67%) started maintenance chemotherapy. Maintenance chemotherapy mainly consisted of the S-1 based regimen. The median duration of continued maintenance chemotherapy was 4 months.

### Overall outcomes

**Figure 1:**
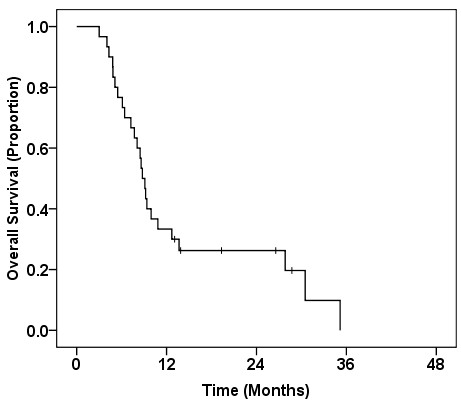
Kaplan-Meier survival curve for overall survival from the start of salvage chemoradiotherapy.

**Figure 2:**
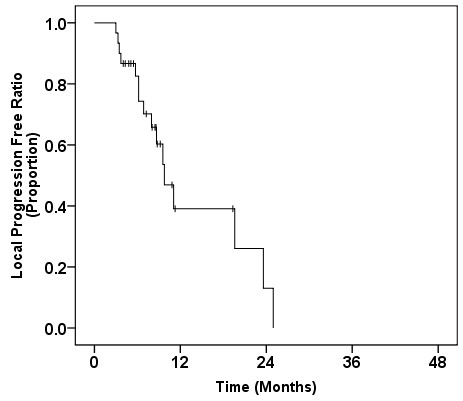
Kaplan-Meier survival curve for the local progression-free ratio from the start of salvage chemoradiotherapy.

The median overall survival time (MST) of the entire patient population from the start of salvage CRT was 8.8 (95% CI, 7.8-9.8) months. The 6 month, 1-year and 2-year survival rates from the start of salvage CRT were 76.7%, 33.3% and 26.3%, respectively (Figure 1). At the time of analysis, four patients were still alive, while 26 patients had died of disease progression. No patients underwent radical resection of their pancreatic cancer after CRT. The median progression-free survival (PFS) time from the start of salvage CRT was 4.9 (95% CI, 3.4-6.3) months. The 6 month, 1-year and 2-year PFS rates were 40.0%, 15.2% and 5.7%, respectively. Sites of disease progression after CRT were documented in all 28 patients with progression; they are summarized in Table 5. The sites of first failure after CRT included distant metastases in 17 patients (61%) and locoregional progression in 10 patients (36%); one patient (3%) had both sites of first failure after CRT. Although prophylactic nodal irradiation was not undertaken, isolated nodal recurrence as a first site of recurrence was observed in only one patient. The median local progression-free time from the start of CRT was 9.8 (95% CI, 7.2-12.3) months (Figure 2). The 6 month, 1-year and 2-year local progression-free rates were 82.5%, 39.1% and 13.0%, respectively. The median distant metastasis-free time from the start of CRT was 6.2 (95% CI: 2.6-9.8) months.

**Table 5:** Sites of first disease progression after salvage chemoradiotherapy

**Disease site**	**No. of patients**	**% patients**
None	2	7
Distant metastases	17	57
Liver	12	
Peritoneum	2	
Liver and peritoneum	1	
Lung	1	
Liver and lung	1	
Locoregional progression	10	33
Local progression	9	
Regional lymph node	1	
Local progression and distant metastases	1	3
Local and peritoneum	1	

In two patients, the primary tumors showed no response to primary chemotherapy and they had PD (Table 2). The primary tumors of these two patients remained stable at the completion of CRT. One patient was not evaluated further because lung metastases emerged at the completion of CRT. She received best supportive care owing to her poor general condition. The primary tumor in the other patient remained stable for 9.6 months, then progressed locally. Both patients died of primary disease at 4.0 and 13.7 months after the start of CRT.

**Figure 3:**
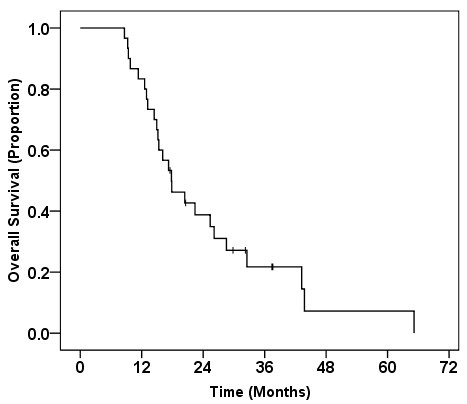
Kaplan-Meier survival curve for overall survival from the start of primary chemotherapy.

Considered overall, the MST from the start of primary chemotherapy was 17.8 (95% CI, 12.3-23.3) months. The 1-, 2-, 3- and 4-year survival rates from the commencement of first-line chemotherapy were 83.3%, 38.8%, 21.7% and 7.2%, respectively (Figure 3).

### Univariate and multivariate analysis of pre-CRT factors influencing survival after CRT

Univariate analysis was performed on 11 different variables to evaluate their potential value in terms of survival after salvage CRT (Table 6). Significant prognostic factors for improved survival included KPS (≥ 80; *p* = 0.022); number of regimens of primary chemotherapy (single; *p* = 0.006); pre-CRT tumor diameter ≤ 4 cm (*p* = 0.04); and pre-CRT serum CA19-9 level (≤ 1000 U/ml; *p* = 0.002). The absence of local progression before salvage CRT (*p* = 0.15) and concomitant use of S-1 during salvage CRT (*p* = 0.09) were not significant prognostic factors. The time from the start of primary chemotherapy to salvage CRT was not associated with survival (*p* = 0.73). Using multivariate analysis, a lower pre-CRT serum CA-19-9 level (≤ 1000 U/ml; *p* = 0.009) and a single regimen of primary chemotherapy (*p* = 0.004) were found to be independent prognostic factors for survival after salvage CRT (Table 7).

**Table 6:** Results of univariate analysis of survival after salvage chemoradiotherapy

** Factors**	**No. of patients**	**Median survival time (months)**	**6-month survival (%)**	**1-year survival (%)**	**2-year survival (%)**	***p*-value**
All patients	30	8.8	77	33	26	
Age						
< 65	14	8.1	79	29	14	
≥ 65	16	9.2	75	38	38	0.2
Gender						
Male	16	8.1	75	31	25	
Female	14	9.2	79	36	29	0.6
Karnofsky performance status						
≥ 80	28	9.1	79	36	28	
< 80	2	4.8	50	0	0	0.03
Primary tumor location						
Head	15	9.4	93	40	33	
Body / tail	15	8.5	60	27	18	0.5
Number of regimens of primary chemotherapy						
1	25	9.4	80	40	32	
2	5	6.1	60	0	0	0.006
Best response to primary chemotherapy						
PR	9	9.2	89	33	33	
SD or PD	21	8.5	71	33	24	0.6
Pre-chemoradiotherapy tumor diameter (cm)						
≤ 4	12	10.8	83	50	50	
> 4	18	8.5	72	22	0	0.04
Pre-chemoradiotherapy serum CA19-9 level (U/ml)						
≤ 1,000	29	10.8	90	47	42	
> 1,000	11	6.4	54	9	0	0.002
Local progression before starting chemoradiotherapy						
Absent	4	NA	80	60	60	
Present	26	8.8	76	28	19	0.15
Time from the start of primary chemotherapy to chemoradiotherapy						
≤ 6 months	12	8.5	75	33	25	
> 6 months	18	8.8	78	33	28	0.9
Combined chemoradiotherapy agents						
5-FU	14	7.2	64	21	14	
S-1	16	9.9	88	44	37	0.09

*PR* partial response, *SD* stable disease, *PD* progressive disease, *NA* not available.

**Table 7:** Results of multivariate analysis of survival after salvage chemoradiotherapy

** Variables**	**Factors**	**Hazard rate (95% CI)**	***p*-value**
Pre-chemoradiotherapy serum CA19-9 level (U/ml)	≤ 1000 versus > 1000	1	0.009
		4.38 (1.45-13.22)	
Number of regimens of primary chemotherapy	1 versus 2	1	0.004
		6.28 (1.78-22.18)	
Local progression before chemoradiotherapy	absent versus present	1	0.6
		1.58 (0.34-7.18)	
Pre-chemoradiotherapy tumor diameter (cm)	≤ 4.0 versus > 4.0	1	0.9
		1.11 (0.35-3.46)	

## Discussion

In the present study, the MST of the entire patient population from the start of salvage CRT was 8.8 months. The median time to local progression from the commencement of salvage CRT was 8.9 months. Before starting CRT, all of the patients experienced failure of the primary chemotherapy. However, the MST of 8.8 months for this cohort is comparable to the historical MST achieved after primary CRT combined with 5-FU [2,14,19]; the median time to local progression was also similar [13]. In addition, the frequency of grade 3–4 non-hematological toxicity observed in the current study was also similar to that reported in previous studies. These findings show that CRT combined with S-1 or 5-FU had moderate anti-tumor activity and an acceptable toxicity profile in patients with LAPC, even after failure of GEM-based primary chemotherapy.

In the literature, the representative MST of patients with LAPC who were included in prospective clinical trials was reported to be 8.4-11.4 months for 5-FU-based CRT [2,3,14,19], 9.2-15.0 months for GEM monotherapy [15,20] and 10.3-11.1 months for GEM-based CRT [20,21]. Generally, only a few patients with LAPC survive for 3 years or more. The MST from salvage CRT in our cohort seems to be inferior to those reported in recent studies involving primary therapy for LAPC. However, if we consider primary chemotherapy and salvage CRT as a combined treatment strategy, the MST of 17.8 months from the start of primary chemotherapy is a promising result. Additionally, long-term survivors from the start of primary chemotherapy in our cohort seem to be distinct, with 22% achieving a 3-year overall survival. In our cohort, only patients who underwent primary chemotherapy and progressed locally without distant metastases were selected to receive salvage CRT. Because of the strong selection bias, we should not compare this outcome to that of prospective clinical trials in the literature. However, the existence of long-term survivors in our cohort suggests that salvage CRT should have some benefit in selected patients with LAPC, even after failure of the primary chemotherapy. The strategy of using chemotherapy alone as a primary treatment for LAPC, followed-by CRT for salvage intent, should be further investigated in prospective clinical trials.

Combined with radiotherapy, S-1 has been demonstrated to exert a synergistic effect against 5-FU-resistant cancer xenografts [22]. We previously conducted a phase I trial to determine the maximum tolerated dose of S-1 with concurrent radiotherapy for LAPC [4]. This dose was 80 mg/m^2^/day, which is the same as the full dose of S-1 when administered alone. The toxicity of CRT combined with S-1 for LAPC was generally mild and manageable with conservative treatment. Several phase II clinical trials of CRT combined with S-1 for LAPC achieved MSTs in the range 14.3-16.2 months [7,8]. These MSTs compare favorably with the historical MSTs reported for CRT combined with 5-FU of 8.4-11.4 months [2,14]. In the current study, either S-1 or 5-FU was combined with radiotherapy. Univariate analysis of survival after subsequent CRT showed a non-significant trend towards better results when CRT was combined with S-1 (Table 6). The occurrence of grade 3–4 non-hematological toxicity during and after CRT was less frequent among the patients who had received CRT combined with S-1, as compared with 5-FU (6% versus 43%). Because of the retrospective nature of this study, a difference in baseline characteristics may inhibit a fair comparison between the two agents. Although a direct comparison between S-1 and 5-FU has not yet been undertaken in a prospective clinical trial, CRT combined with S-1 is an attractive alternative to 5-FU-based CRT.

The value of S-1 in pancreatic cancer is not limited to its sensitizing effect during CRT. Single agent S-1 has excellent activity regarding chemo-naïve metastatic pancreatic cancer, with a response rate of 37.5% and a MST of 9.2 months [23]. S-1 is the first agent that has not proved inferior to GEM as a single agent for the treatment of advanced pancreatic cancer in a phase III randomized-controlled trial [16]. S-1 also retains its activity in relation to advanced pancreatic cancer even after the failure of GEM, with a response rate of 21% [24]. Accordingly, in the current study, the activity of salvage CRT with S-1 should be related to the excellent systematic effect of the agent on subclinical distant metastasis, as well as its local sensitizing effect.

Recently, induction chemotherapy has become a major component in the treatment strategy for LAPC. Two well-designed retrospective studies have shown that induction chemotherapy followed by CRT yielded a survival benefit over primary CRT or continued chemotherapy alone for LAPC [12,25]. More recently, several phase II prospective clinical trials have been conducted to evaluate the value of induction chemotherapy followed by CRT, which resulted in MSTs in the range 12.6-19.2 months [26-28]. The optimum duration of induction chemotherapy for LAPC continues to be a matter of debate. Recent prospective clinical trials that included induction chemotherapy for LAPC had chosen to evaluate the effects of 2–6 months of induction therapy [26-28]. In the current study, the median duration of primary chemotherapy was 7 months, which is longer than those used in these prospective trials. Because patients with rapidly progressing occult-metastatic disease were excluded from the present study, the tumors in our cohort might have deviated to relatively chemo-responsive tumors. Therefore, the duration of primary chemotherapy was not associated with survival after CRT in the current study. We could not draw any conclusion with regard to the optimum duration of induction chemotherapy from this retrospective cohort study.

In agreement with the current study, previous studies have shown that a highly-elevated CA 19–9 level is a poor prognostic factor for patients who had received CRT for LAPC [29,30]. A highly elevated serum CA19-9 level in patients prior to CRT suggests chemo-resistance of the tumor, as well as the existence of progressive occult metastasis. These patients might gain little benefit from the addition of salvage CRT.

Multivariate analysis revealed that the use of two regimens of primary chemotherapy was an unfavorable factor for survival after CRT. The MST of the patients who received two regimens of primary chemotherapy was 6.1 months from the start of salvage CRT, and no patient survived for 12 months or longer thereafter (Table 6). In all of the patients (n = 5) who underwent two regimens of primary chemotherapy before CRT, S-1 was used as a second-line chemotherapy. Of these patients, three received salvage CRT combined with 5-FU, and two received salvage CRT combined with S-1. Because both 5-FU and S-1 are fluorinated pyrimidine agents, failure of the tumor to respond to treatment with S-1 should cause resistance to salvage CRT combined with either 5-FU or S-1. If there are any signs of failure to respond to the primary chemotherapy, without distant metastasis, salvage CRT could be a treatment of choice as a second-line therapy.

Because of the retrospective nature of the current study, there were a number of limitations that affected the interpretation of our findings. The number of patients was very limited and the patient population was not homogeneous because of different clinical backgrounds, and they received CRT with salvage intent. Also, the patients were collected for over a period of 7 years, non-consecutively. The clinical response to primary chemotherapy was generally better than previously reported, possibly because of the exclusion of patients with chemo-resistant occult distant metastasis. Only patients who underwent primary chemotherapy and progressed locally without distant metastases were selected and included in the current analysis.

Whether or not the addition of chemotherapy prior to CRT will contribute to prolonging the survival of patients with LAPC has not been elucidated with sufficient statistical power in a prospective clinical trial. We are now investigating the value of induction chemotherapy with GEM versus no induction chemotherapy for LAPC in a multi-institutional randomized phase II study involving S-1 and concurrent radiotherapy (JCOG1106, UMIN000006811). A future phase III study will be conducted to compare GEM monotherapy and S-1 based CRT with or without induction GEM, depending on the results of the JCOG1106 study. Another phase III study, the GERCOR LAP 07 phase III trial (http://www.clinicaltrials.gov, identifier code NCT00634725) is also ongoing. This study was designed to elucidate the benefit of induction chemotherapy followed by CRT combined with capecitabine, with or without erlotinib during induction chemotherapy and a CRT phase. In future, results from these prospective clinical trials will become available to further define the role of chemotherapy followed by CRT for LAPC.

## Conclusions

CRT combined with S-1 or 5-FU had moderate anti-tumor activity in patients with LAPC even after failure of GEM-based primary chemotherapy. If there are any signs of failure to primary chemotherapy without distant metastasis, salvage CRT could be a treatment of choice as a second-line therapy. Patients with a relatively low serum CA19-9 level after primary chemotherapy may obtain additional survival benefit from salvage CRT. The strategy of using chemotherapy alone as a primary treatment for LAPC, followed-by CRT with salvage intent should be further investigated in prospective clinical trials.

## Competing interests

The authors declare that they have no competing interests.

## Authors’ contributions

HM, YI and JI participated in the design of the study, performed the statistical analysis, interpretation of data, and drafted the manuscript. HM, YI, NM, MM and MS carried out the chemoradiotherapy and analyzed tumor response. CM, HU, TO and SK carried out the chemotherapy and analyzed tumor response. All of the listed authors contributed to the writing of the manuscript. All authors read and approved the final manuscript.

## Pre-publication history

The pre-publication history for this paper can be accessed here:

http://www.biomedcentral.com/1471-2407/12/609/prepub
